# First case report of lorlatinib in the treatment of ALK-fusion-positive lung adenocarcinoma with ovarian metastasis: Clinicopathological and molecular characterization

**DOI:** 10.1097/MD.0000000000045222

**Published:** 2025-10-31

**Authors:** Tianxiang Xu, Shiyu Hua, Jingying Wang, Mengqi Wu, Xinyi Qiu, Jiamin Hong, Yu Zhang, Keding Shao, Jue Wang

**Affiliations:** a Department of Oncology, Hangzhou Traditional Chinese Medicine Hospital Affiliated to Zhejiang Chinese Medical University, Hangzhou, China; b Department of Radiology, The Third Affiliated Hospital of Zhejiang Chinese Medical University, Hangzhou, China; c Department of Nephropathy, Hangzhou Traditional Chinese Medicine Hospital Affiliated to Zhejiang Chinese Medical University, Hangzhou, China; d Department of Oncology, The First Affiliated Hospital of Zhejiang Chinese Medical University (Zhejiang Provincial Hospital of Traditional Chinese Medicine), Hangzhou, China; e Department of Pathology, Hangzhou Traditional Chinese Medicine Hospital Affiliated to Zhejiang Chinese Medical University, Hangzhou, China; f Department of Scientific Research, Zhejiang Chinese Medical University, Hangzhou, China.

**Keywords:** anaplastic lymphoma kinase fusion (ALK-fusion), case report, lorlatinib, ovarian metastasis

## Abstract

**Background:**

Ovarian metastasis from anaplastic lymphoma kinase (ALK)-positive lung adenocarcinoma is exceedingly rare. Reporting such cases is vital for understanding its clinical management.

**Patient concerns:**

A 49-year-old woman presented with a 10-day history of amenorrhea and lower abdominal pain.

**Diagnoses:**

Imaging revealed bilateral ovarian masses and liver nodules. Liver biopsy and genetic testing confirmed the diagnosis of stage IV EML4-ALK fusion-positive lung adenocarcinoma with ovarian and hepatic metastases.

**Interventions:**

First-line treatment with the third-generation ALK inhibitor lorlatinib (100 mg, once daily) was initiated.

**Outcomes:**

The patient achieved a partial response with significant lesion regression and normalized tumor markers, sustained for over 12 months. No severe adverse events occurred.

**Lessons:**

This case underscores the importance of molecular profiling in diagnosing metastatic tumors and confirms lorlatinib as a highly effective first-line therapy for ALK-positive non-small cell lung cancer with atypical metastases.

## 1. Introduction

Lung cancer ranks as one of the most prevalent and lethal malignancies worldwide.^[1]^ While fewer than 5% of lung cancer cases metastasize to uncommon sites, ovarian metastasis occurs in merely 0.07% of cases.^[2]^ The EML4-ALK-fusion is detected in only 3% to 5% of non-small cell lung cancer (NSCLC) patients.^[3]^ Consequently, EML4-ALK-fusion-positive lung adenocarcinoma with ovarian metastasis represents an exceptionally rare clinical scenario. Importantly, the presence of EML4-ALK rearrangement provides an optimal therapeutic target for advanced-stage patients. Herein, we present a CARE checklist-compliant case report documenting an EML4-ALK-fusion-positive lung adenocarcinoma patient with ovarian metastasis who demonstrated significant tumor regression and prolonged survival following lorlatinib treatment.

## 2. Case presentation

A 49-year-old Chinese female presented to our hospital on May 7, 2024, with a 10-day history of amenorrhea and lower abdominal pain. She reported a 5-year history of right ovarian physiological cysts, with regular annual gynecological ultrasound follow-up; the last follow-up in December 2023 showed a cyst diameter of approximately 2.3 cm without abnormal enlargement. Her menstrual cycle had been regular (28–30 days) with moderate flow and no dysmenorrhea over the past year, and this 10-day amenorrhea was her first episode of menstrual irregularity. Enhanced CT imaging at the Women’s Hospital of Zhejiang University School of Medicine demonstrated: Bilateral complex adnexal masses (cystic-solid components), Multiple hepatic nodules, Enlarged para-aortic lymph nodes, radiological suspicion of: Bilateral ovarian malignancy and Hepatic metastases. Subsequent comprehensive diagnostic evaluation at our institution revealed significantly elevated tumor markers (CEA 52.50 ng/mL, CA-125 748.54 U/mL, CA724 36.05 IU/mL), pelvic ultrasound demonstrating a 12.1 × 10.2 × 7.6 cm fused pelvic mass, chest CT showing multiple pulmonary nodules including a basal segment nodule in the right lower lobe, abdominal MRI identifying multiple hepatic nodules and enlarged retroperitoneal lymph nodes suggestive of metastatic disease, contrast-enhanced ultrasound of the liver revealing a mass with peripheral rim enhancement and central hypo-enhancement, while gastrointestinal endoscopy showed no significant abnormalities.

**Figure 1.**
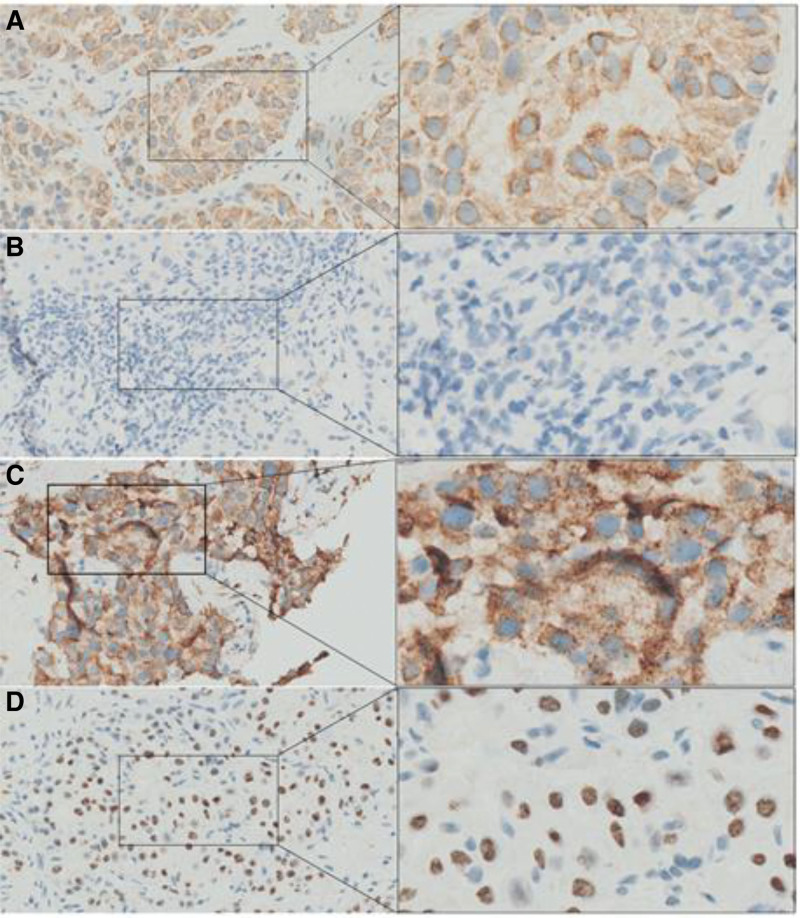
Immunohistochemical staining of liver biopsy specimens. (A) CK7 immunopositivity in tumor cells (IHC, ×400). (B) CK20 immunonegativity in tumor cells (IHC, ×400). (C) NapsinA immunopositivity in tumor cells (IHC, ×400). (D) TTF-1 immunopositivity in tumor cells (IHC, ×400).

The liver biopsy results on May 16, 2024 indicated the presence of adenocarcinoma infiltration or metastasis, with immunohistochemical findings consistent with pulmonary adenocarcinoma (Fig. 1), demonstrating positive staining for NapsinA, CK7, CK19, and TTF-1, focal positivity for P16, moderately positive P53 expression, and a Ki67 proliferation index of approximately 10%, while being negative for CK20, ER, and partial response. Subsequent NGS analysis on May 22, 2024, identified an EML4-ALK-fusion mutation (EML4:exon19–ALK:exon20), confirming the final diagnosis of EML4-ALK-fusion-positive pulmonary adenocarcinoma with ovarian and hepatic metastases, clinically staged as cT1N0M1 (Stage IV).

**Figure 2.**
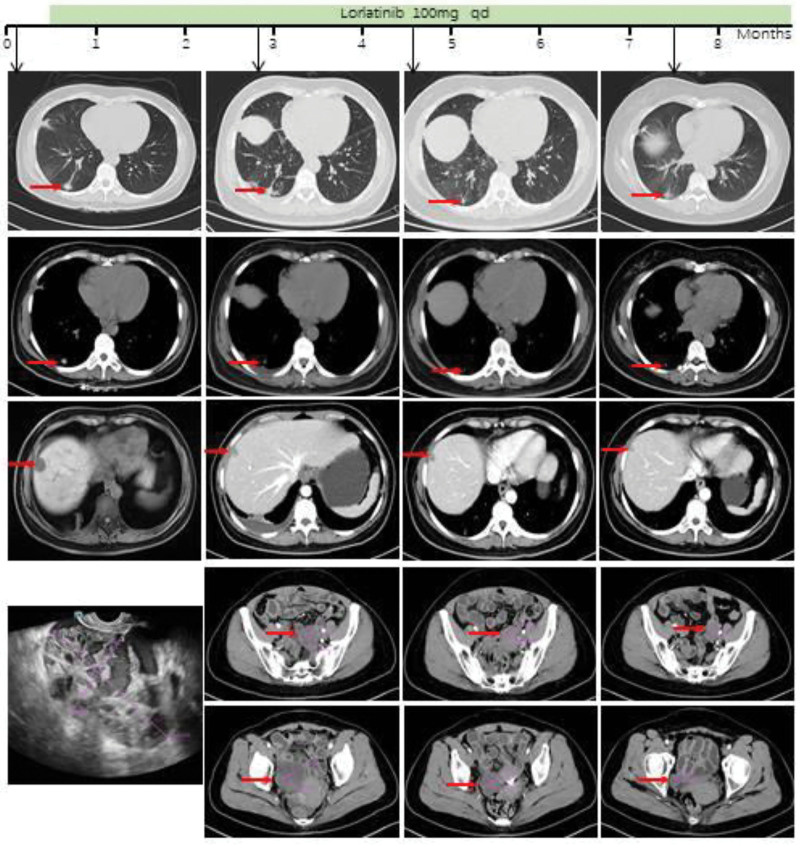
Treatment Timeline and Duration. Illustrates of the treatment received by the patient (the green bars), the corresponding PFS at the time of hospitalization (the black mark), and chest and abdominal CT assessments before and after treatment (black arrows indicating pre-hospitalization and quarterly follow-up times, The first row shows lung window images, the second row shows mediastinal window images, the third row shows the right ovary, the fourth row shows the left ovary, and separate images are provided for ovarian ultrasound. The red arrows indicate the tumor masses.). CT = computed tomography, PFS = progression-free survival, Qd = once daily.

**Figure 3.**
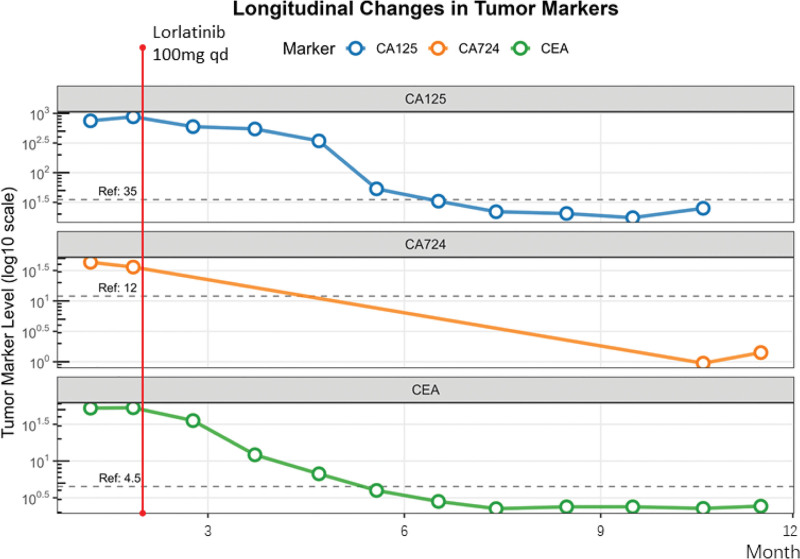
CA-125 (blue curve) and CEA (green curve): Serum levels decreased to a stable plateau by 6 months posttreatment, indicating an early rapid therapeutic response. CA724 (orange curve): Gradual reduction to the detection limit was observed.

Based on genetic testing, lorlatinib (100 mg once daily, qd) was initiated on May 28, 2024. Quarterly contrast-enhanced chest-abdominal CT scans demonstrated progressive reduction of tumor burden in ovarian, hepatic, and pulmonary lesions (Fig. 2). Serial tumor marker monitoring over 12 months revealed rapid decline below baseline levels with sustained stabilization, consistent with therapeutic efficacy (Fig. 3). Per RECIST 1.1 criteria, the patient achieved partial response (Table 1).

**Table 1:** Timeline.

Date	Time point	Event and key details
April 28, 2024	Onset of symptoms	Unexplained amenorrhea occurs
May 7, 2024	First visit and initial workup	Admitted to the oncology department; increased tumor markers (CEA 52.50 ng/mL, CA-125 748.54 U/mL, CA724 36.05 IU/mL), pelvic ultrasound showed a 12.1 × 10.2 × 7.6 cm fused pelvic mass; chest CT showed multiple pulmonary nodules; abdominal MRI showed multiple hepatic nodules and enlarged retroperitoneal lymph nodes suggestive of metastatic disease; contrast-enhanced ultrasound of the liver showed a mass with peripheral rim enhancement and central hypo-enhancement; gastrointestinal endoscopy showed no significant abnormalities.
May 16, 2024	Definitive diagnosis	A liver biopsy performed revealed adenocarcinoma infiltration or metastasis, with immunohistochemical findings consistent with pulmonary adenocarcinoma
May 22, 2024	Perfect inspection.	NGS: EML4-ALK-fusion-positive
May 28, 2024	Intervention	Lorlatinib (100 mg po qd)
June 13, 2024	Symptom relief	The abdominal pain has improved significantly
July 11, 2024, July 23, 2024	Adverse reaction and management	Treatment-emergent hyperlipidemia and medicamentous liver lesion, Rosuvastatin and compound glycyrrhizin tablet
September 18, 2024	5th follow-up (inpatient)	Imaging studies indicate that the tumor has been significantly controlled, per RECIST 1.1 criteria, the patient achieved partial response
November 13, 2024	14th follow-up (outpatient)	The serum levels of tumor marker decreased to a stable plateau by 6 mo posttreatment

CT = computed tomography.

## 3. Discussion

NSCLC most frequently metastasizes to the brain, followed by bone and liver, with other organ involvement occurring in <5% of cases. Among ovarian malignancies, secondary metastatic tumors account for approximately 4.2%,^[4]^ of which 60% to 80% originate from gastrointestinal (commonly termed Krukenberg tumors) and breast carcinomas, while pulmonary metastases represent an exceptionally rare occurrence. Furthermore, the largest case series study of pulmonary adenocarcinoma with ovarian metastases^[5]^ demonstrated that anaplastic lymphoma kinase (ALK) rearrangements – particularly EML4-ALK fusions – represent the predominant molecular alteration in pulmonary malignancies metastasizing to the ovaries, with a striking predilection for young, never- or light-smoking East Asian women. This case of EML4-ALK + lung adenocarcinoma with ovarian metastasis is extremely rare and provides valuable insights into the pathological, molecular research, and treatment strategies for such patients.

The diagnosis of pulmonary adenocarcinoma with ovarian metastases presents significant clinical challenges, as highlighted by Julie, whose study demonstrated that 16% of ovarian involvement may be detected prior to identification of the primary lung tumor.^[6]^ This case illustrates this diagnostic dilemma. Initially, only bilateral ovarian masses were found, while no significant abnormalities were found on chest CT, leading to the primary consideration of ovarian neoplasia. This indicates that the primary and metastatic ovarian masses are difficult to distinguish clinically. Firstly, abdominal and pelvic pain represent the most frequent symptomatic manifestations of secondary ovarian tumor. Secondly, imaging features and immunohistochemical staining are very useful in distinguishing between primary and metastatic ovarian tumors. Contemporary multicenter research^[7]^ confirms that metastatic ovarian tumors predominantly present as bilateral masses (<10 cm diameter), with multiple studies^[8,9]^ establishing these characteristics as the strongest predictive features for metastatic origin - findings concordant with our patient’s bilateral ovarian involvement (initial sizes: 6.2 cm and 5.8 cm). The diagnostic IHC profile (CK7+/CK20-) exhibited 86.7% specificity for pulmonary origin in our institutional cohort (2018–2023, n = 47), consistent with literature reports that > 80% of lung-derived adenocarcinomas demonstrate this pattern.^[10]^ Notably, the combination of NapsinA and TTF-1 immunopositivity provided superior discriminatory capacity (sensitivity 92.1%, specificity 94.3%) for identifying pulmonary-derived metastases,^[11]^ as confirmed in this case’s liver biopsy specimens. This patient initially presented with nonspecific abdominal pain in the absence of classic pulmonary adenocarcinoma symptoms. Diagnostic imaging identified bilateral ovarian masses (5–6 cm diameter), while immunohistochemical analysis revealed a CK7(+)/CK20(−)/NapsinA(+)/TTF-1(+) profile - findings fully concordant with both radiographic predictors and diagnostic criteria for pulmonary adenocarcinoma with ovarian metastases. Histopathological verification through biopsy remains the gold standard for discriminating between primary and metastatic malignancies in preoperative settings. In this diagnostically challenging case, where thoracic and abdominopelvic imaging yielded inconclusive results, ultrasound-guided liver biopsy provided definitive confirmation of pulmonary origin due to differences in survival rates and treatment approaches, distinguishing between primary and secondary ovarian malignancies is of significant importance in determining subsequent treatment strategies and prognosis for patients.

Lorlatinib demonstrates superior efficacy in inhibiting resistant mutations and delaying central nervous system metastasis compared to other ALK tyrosine kinase inhibitors (ALK-TKIs). Three interim analyses of the CROWN trial with median follow-up data,^[12]^ revealed that lorlatinib achieved an unprecedented median progression-free survival (mPFS) exceeding 60 months (95% CI: 64.3-NR). This represents the longest mPFS observed with any single-agent molecular targeted therapy in advanced NSCLC and across all metastatic solid tumors, establishing its optimal candidacy as a first-line treatment strategy for treatment-naïve NSCLC patients. Patients with ALK-positive NSCLC are more likely to develop brain metastases compared to those with RET or ROS1 rearrangements.^[13]^ Lorlatinib significantly improves PFS in patients with or without baseline brain metastases, and more than half of central nervous system adverse events (AEs) resolve spontaneously without intervention or dose adjustment.^[12]^ These findings indicate that lorlatinib has a clear advantage over other ALK inhibitors in delaying or preventing brain metastases. The patient’s head MRI showed transient linear meningeal enhancement in the right frontal lobe. After our team’s assessment, we believed that lorlatinib, due to its superior blood-brain barrier penetration and lower mutation rate of drug resistance, is more suitable to be used as the first-line treatment option. Follow-up cranial MRI examinations from September to December revealed complete resolution of the initial linear enhancement in the right frontal meninges, further confirming lorlatinib pronounced efficacy in both preventing and treating brain metastases. We considered that the Lorlatinib-associated AEs were mainly grade 1/2. The most common ones were hypertriglyceridemia, hypercholesterolemia and weight gain. Therefore, at the beginning of the treatment, we prophylactically used statins to alleviate these metabolic complications. Meanwhile, this patient already had liver metastasis and the risk of future liver dysfunction was considered. Eventually, we decided to choose rosuvastatin as the intervention measure.

Patients with metastatic ovarian cancer exhibit significantly lower 5-year survival rates compared to those with primary ovarian cancer, and oophorectomy demonstrates limited impact on prognosis.^[14]^ Furthermore, ALK-positive NSCLC is characterized by highly aggressive biological behavior, contributing to poor clinical outcomes. However, the presence of the EML4-ALK-fusion target may justify the use of lorlatinib, which could substantially prolong patient survival duration to a clinically meaningful extent. A study investigating lorlatinib efficacy across EML4-ALK variants^[15]^ demonstrated that both V1 and V2 subtypes achieved median PFS (mPFS) that was not reached, while V3 exhibited an mPFS of 33.3 months. In the present case, NGS analysis revealed a novel EML4 (exon19)-ALK (exon20) fusion subtype not previously documented in the literature. This successful lorlatinib treatment strategy provides valuable therapeutic reference for managing this rare EML4-ALK variant in NSCLC patients. To optimize survival outcomes in female patients with ovarian metastases, clinicians should maintain a high index of suspicion for lung cancer-derived metastases. A comprehensive diagnostic approach incorporating histopathological biopsy and driver gene testing is essential for accurate molecular subtyping. For patients without surgical indications, optimal systemic therapy – particularly targeted treatment – should be prioritized as first-line management.

Furthermore, given the common limitations of targeted drug resistance, immune checkpoint inhibitors(ICIs) should be incorporated into the treatment regimen as early as possible. A meta-analysis on ICIs^[16]^ demonstrated that adjuvant therapy with PD-1/PD-L1 inhibitors may significantly reduce the risk of recurrence Another study^[17]^ investigating AEs associated with ICIs revealed that immunotherapy may confer greater clinical benefits in female patients, who concurrently demonstrate a higher incidence of hypothyroidism. In future outpatient follow-up for this patient, more personalized monitoring and management strategies could potentially be incorporated.

## 4. Limitations

The selection of appropriate therapeutic agents requires careful consideration of each ALK-TKI’s toxicity profile, along with patient comorbidities and preferences. Given that this study included only 1 case of lung adenocarcinoma with ovarian metastasis and had a follow-up period of less than 1 year, the long-term prognosis cannot be reliably assessed. Furthermore, the extremely limited sample size and absence of contemporaneous control cases preclude meaningful comparison of the therapeutic efficacy between different treatment regimens.

## 5. Conclusion

Ovarian metastases originating from pulmonary adenocarcinoma are exceptionally rare. We present a case of a middle-aged female patient with EML4-ALK-positive pulmonary adenocarcinoma and ovarian metastases. The clinical application of lorlatinib provides an effective therapeutic option for ALK-positive patients. Significant controversy persists in clinical practice regarding whether the optimal treatment strategy for metastatic ALK-positive NSCLC should initiate with second-generation ALK-TKIs or proceed directly to third-generation TKIs as first-line therapy. This case may represent the first clinical evidence suggesting lorlatinib potential efficacy in treating EML4-ALK + pulmonary adenocarcinoma with ovarian metastases. We hope this case report will provide evidence supporting the use of targeted agents in EML4-ALK-fusion pulmonary adenocarcinoma patients with ovarian metastases and stimulate novel research directions in EML4-ALK targeted therapy. This case may represent a unique clinical scenario. The targeted therapy selection strategy described herein should be considered exploratory. Further investigation with larger datasets remains imperative.

## Author contributions

**Conceptualization:** Mengqi Wu, Jiamin Hong.

**Data curation:** Tianxiang Xu, Shiyu Hua.

**Resources:** Jingying Wang.

**Software:** Yu Zhang.

**Visualization:** Xinyi Qiu.

**Writing – original draft:** Tianxiang Xu, Shiyu Hua.

**Writing – review & editing:** Keding Shao, Jue Wang.

## References

[1] Bray F Laversanne M Sung H. Global cancer statistics 2022: GLOBOCAN estimates of incidence and mortality worldwide for 36 cancers in 185 countries. CA Cancer J Clin. 2024;74:229–63. 38572751 10.3322/caac.21834

[2] Niu FY Zhou Q Yang JJ. Distribution and prognosis of uncommon metastases from non-small cell lung cancer. BMC Cancer. 2016;16:149. 26911831 10.1186/s12885-016-2169-5 PMC4766662

[3] Vašíková A. [EML4-ALK fusion gene in patients with lung carcinoma: biology, diagnostics and targeted therapy]. Klinicka Onkologie. 2012;25:434–9. 23301645

[4] Timmerman D Van Calster B Testa A. Predicting the risk of malignancy in adnexal masses based on the simple rules from the international ovarian tumor analysis group. Am J Obstet Gynecol. 2016;214:424–37. 26800772 10.1016/j.ajog.2016.01.007

[5] Bi R Bai Q Zhu X. ALK rearrangement: a high-frequency alteration in ovarian metastasis from lung adenocarcinoma. Diagn Pathol. 2019;14:96. 31455365 10.1186/s13000-019-0864-7 PMC6712650

[6] Irving JA Young RH. Lung carcinoma metastatic to the ovary: a clinicopathologic study of 32 cases emphasizing their morphologic spectrum and problems in differential diagnosis. Am J Surg Pathol. 2005;29:997–1006. 16006793

[7] Cai SQ Wu MR Ma XL. Mucin-producing tumors of the ovary——preoperative differentiation between metastatic ovarian mucinous carcinoma and primary mucinous malignant tumors. J Ovarian Res. 2024;17:59. 38481236 10.1186/s13048-024-01382-8 PMC10936019

[8] Seidman JD Kurman RJ Ronnett BM. Primary and metastatic mucinous adenocarcinomas in the ovaries: incidence in routine practice with a new approach to improve intraoperative diagnosis. Am J Surg Pathol. 2003;27:985–93. 12826891 10.1097/00000478-200307000-00014

[9] Khunamornpong S Suprasert P Pojchamarnwiputh S Na Chiangmai W Settakorn J Siriaunkgul S. Primary and metastatic mucinous adenocarcinomas of the ovary: evaluation of the diagnostic approach using tumor size and laterality. Gynecol Oncol. 2006;101:152–7. 16300822 10.1016/j.ygyno.2005.10.008

[10] Chu P Wu E Weiss LM. Cytokeratin 7 and cytokeratin 20 expression in epithelial neoplasms: a survey of 435 cases. Modern Pathol. 2000;13:962–72. 10.1038/modpathol.3880175 11007036

[11] Li H Chen Y Wang Y. Ovarian metastases from ALK-positive lung adenocarcinoma: a case report and review of the literature. Transl Cancer Res. 2022;11:3391–9. 36237252 10.21037/tcr-22-273 PMC9552264

[12] Solomon BJ Liu G Felip E. Lorlatinib versus crizotinib in patients with advanced ALK-positive non–small cell lung cancer: 5-year outcomes from the phase III crown study. J Clin Oncol. 2024;42:3400–9. 38819031 10.1200/JCO.24.00581 PMC11458101

[13] Drilon A Lin JJ Filleron T. Frequency of brain metastases and multikinase inhibitor outcomes in patients with RET-rearranged lung cancers. J Thor Oncol. 2018;13:1595–601. 10.1016/j.jtho.2018.07.004 PMC6434708 30017832

[14] Li W Wang H Wang J Fangfang LV Zhu X Wang Z. Ovarian metastases resection from extragenital primary sites: outcome and prognostic factor analysis of 147 patients. BMC Cancer. 2012;12:278. 22759383 10.1186/1471-2407-12-278 PMC3487894

[15] Bearz A Martini JF Jassem J. Efficacy of lorlatinib in treatment-naive patients with ALK-positive advanced NSCLC in relation to EML4::ALK variant type and ALK with or without TP53 mutations. J Thor Oncol. 2023;18:1581–93. 10.1016/j.jtho.2023.07.023 37541389

[16] Rizzo A Mollica V Marchetti A. Adjuvant PD-1 and PD-L1 inhibitors and relapse-free survival in cancer patients: the MOUSEION-04 study. Cancers. 2022;14:4142. 36077679 10.3390/cancers14174142 PMC9455029

[17] Vitale E Rizzo A Maistrello L. Sex differences in adverse events among cancer patients receiving immune checkpoint inhibitors: the MOUSEION-07 systematic review and meta-analysis. Sci Rep. 2024;14:28309. 39550353 10.1038/s41598-024-71746-z PMC11569249

